# Host Specificity of *Snodgrassella* in Eastern and Western Honeybees and Its Effects on Naturally Occurring Deformed Wing Virus Titers

**DOI:** 10.3390/insects16050478

**Published:** 2025-05-01

**Authors:** Nihong Zhou, Shangning Yang, Ruike Wei, Fuliang Hu, Dandan Liu, Huoqing Zheng

**Affiliations:** 1 College of Animal Sciences, Zhejiang University, Hangzhou 310058, China; zhou_nh@zju.edu.cn (N.Z.); yangsn@zju.edu.cn (S.Y.); wrk0326@zju.edu.cn (R.W.); flhu@zju.edu.cn (F.H.); hqzheng@zju.edu.cn (H.Z.); 2 Institute of Agricultural Research, Xizang Academy of Agriculture and Animal Husbandry Sciences, Lasa 850032, China

**Keywords:** *Snodgrassella*, *Apis mellifera*, *Apis cerana*, gut symbiont, host specificity, deformed wing virus, immune genes

## Abstract

Honeybee gut microbiota undergoes long-term adaptive evolution with the host, resulting in host-specific genomic and functional characteristics. However, the specific role of host-specialized strains in shaping host physiological functions remains understudied. This study investigates the host specificity of the core gut symbiont *Snodgrassella* in *A. cerana* and *A. mellifera*, exploring its effects on immune response and natural virus suppression through genomic analysis and colonization experiments. Genomic analysis revealed that strain from *A. mellifera*, exhibited a larger genome and greater gene content compared to strain from *A. cerana*. Competitive colonization experiments showed that although strains from different origins had similar colonization efficiency in the host, host-specific strain displayed a clear home-field advantage in the competitive colonization process. Moreover, Inoculation of *A. mellifera* with its native *Snodgrassella* strain significantly reduced Deformed Wing Virus (DWV) titers, whereas a non-native strain had no effect. In contrast, neither strain altered DWV or Sacbrood Virus (SBV) levels in *A. cerana*. Immune gene analysis revealed that only the native *Snodgrassella* strain upregulated *defensin 2* in *A. mellifera*, while no significant changes occurred in *A. cerana* with either strain. These results suggest that *Snodgrassella* exhibits host specificity at the strain level, influencing both host immune response and virus suppression, with non-native strains showing reduced efficacy in these functional roles, especially in *A. mellifera*.

## Simple Summary

The composition of the honeybee gut microbiota is relatively simple, making it an ideal model for studying host-microbe interactions. While the host-specific evolution of gut symbionts in *Apis mellifera* and bumblebees has been well studied, similar research within the *Apis* genus remains limited. This study focuses on the core gut symbiont *Snodgrassella* from two closely related species, *Apis cerana* and *A. mellifera*, to explore how host-specific evolutionary adaptations of the gut microbiota influence the functional development of honeybees.

## 1. Introduction

The digestive tracts of animals harbor complex microbial communities, including bacteria, archaea, and fungi [1]. These gut microbiota provide key functions for the host, such as energy acquisition, metabolism, and immune regulation [2,3]. Additionally, gut microbiota reflects the evolutionary history of the host [4]. Typically, gut microbiota within the same species were more similar to each other than between different species, and interspecies differences in microbial communities are closely linked to the host’s evolutionary divergence. Host-specific microbiota plays a critical role in maintaining host health and facilitating interspecies adaptation [5]. For instance, in rodents, host-specific microbiota is involved in important processes such as pathogen resistance, nutrient utilization, and growth and development [5,6,7]. Compared to mammals, the gut microbiota of eusocial bees is relatively simple and stable [8]. The core microbiota of adult worker bees consists of 8 to 10 bacterial types [9,10,11]. Within these social bee species, the diversity and host specificity of gut microbiota have been demonstrated at the strain level. For example, *Snodgrassella* exhibits high species diversity and broad host adaptability [12]. These clusters exhibit functional differentiation and ecological niche specialization through gene recombination or single nucleotide polymorphisms, forming a nutritional network with other symbiotic bacteria and establishing a close symbiotic relationship with the host [13,14]. The diversity and composition of gut microbiota not only influence the host’s social behavior and task allocation but also drive the adaptive evolution of host species [13,15,16,17]. However, the extent to which gut microbiota from closely related honeybee species contribute differently to host function, particularly in terms of native and non-native strains, remains largely unresolved.

The core lineages of eusocial bees began co-evolving with their hosts around 80 million years ago, diverging from corbiculate bees and gradually developing host-specific structures [18,19,20]. *A. mellifera* and bumblebee share similar distribution patterns and core gut microbiota. Genomic analysis and colonization experiments based on the core gut members *Snodgrassella*, *Gilliamella*, and *Lactobacillus* of these two bee species show that the core bacterial types shared by eusocial bees have evolved into different sister lineages and hold a home-field advantage in host competition [12,20,21,22,23,24]. The colonization advantage of core strains such as *Snodgrassella alvi*, *Gilliamella apicola*, and *Frischella perrara* is closely related to their encoding of the Type VI Secretion System (T6SS) and associated effectors [25,26]. In *S. alvi*, two T6SS systems are present, with T6SS-1 playing a crucial role in regulating competition between same-species bacteria [27]. At the transcriptional level, the host *A. mellifera* activates gut immune pathways to reject colonization by non-native strains (such as those derived from bumblebees), further supporting the adaptive evolution between the host and its gut microbiota, further supporting the adaptive evolution between the host and its gut microbiota [24]. As two closely related species within the genus *Apis*, *A. cerana* and *A. mellifera* show significant strain-level variation in their gut microbiota based on metagenomic sequencing, and functional differentiation is also observed [28,29]. Recent studies have shown that genomic differences between *Gilliamella* strains from *A. cerana* and *A. mellifera* affect functional divergence and influence host transcriptional responses [30]. However, research on the colonization of *Snodgrassella* strains in the gut of *A. cerana* and *A. mellifera*, and the influence of *Snodgrassella* strains from different sources on the physiological processes of *A. cerana*, remains limited. As a native species of Asia, *A. cerana* is a critical pollinator and honey producer in the region. Therefore, an in-depth study of its gut microbiota’s function and evolution, particularly exploring strain variation and host interaction patterns among closely related species, will provide important insights for formulating targeted and sustainable management strategies.

Honeybee health is threatened by various viruses, with DWV and SBV being common asymptomatic infections in *A. mellifera* and *A. cerana*, respectively [31]. DWV is the most widely distributed honeybee virus globally and is transmitted by the *Varroa destructor* mite, making it a major contributor to the increased overwintering mortality of *A. mellifera* colonies [32]. In contrast, SBV poses a significant threat to *A. cerana*, primarily infecting 1-2-day-old larvae and spreading rapidly within colonies [33]. Its impact is particularly pronounced during periods of low nectar and pollen availability, such as early spring and late autumn [34]. Antimicrobial peptide, including apidaecin, abaecin, hymenoptaecin, defensin, royalisin and melittin, play key roles in the immune response to pathogens in honeybee hemolymph [35]. For instance, honeybees upregulate AMP-related gene expression through the modulation of the Toll/Imd signaling pathway to resist viral infections such as DWV and Israeli Acute Paralysis Virus (IAPV) [36,37]. The gut microbiota is crucial in regulating host immune responses and antiviral defenses. Inoculation with a complete gut microbiota has been shown to significantly reduce *A. cerana* mortality from *Nosema ceranae* infection and upregulate the expression of *apidaecin*, *abaecin*, and *hymenoptaecin* genes [38]. Furthermore, core symbionts, such as *S. alvi*, have been shown to stimulate the expression of *apidaecin* and *hymenoptaecin* genes, thereby enhancing honeybee resistance to DWV infection [39]. However, studies on whether the regulation of viral titers by gut microbiota varies due to the host origin remain limited. Therefore, further investigation of the regulation of viral titers by honeybee gut microbiota from different host origins will provide important insights into host-microbe co-evolution and its impact on honeybee health.

In this study, genomic analysis, and colonization experiments were conducted using gut *Snodgrassella* isolates from *A. cerana* and *A. mellifera* to validate the host-specific evolution of gut microbiota between closely related hosts. The study also evaluated the impact of strain differences on naturally occurring viral titers and immune gene expression, with the aim of revealing the effects of gut microbiota strain variation on host physiology and further elucidating the adaptive evolution pattern between hosts and their gut microbiota.

## 2. Materials and Methods

### 2.1. Strain Cultivation and DNA Extraction

At the experimental apiary of Zhejiang University, 10 forager bees were collected from each of the two honeybee species, *A. cerana* and *A. mellifera*. Both honeybee colonies were kept in the same apiary and had not been treated with antibiotics. Specifically, no pharmaceutical interventions were used in the management of *A. cerana*, while *A. mellifera* colonies were only treated with routine acaricides for mite control. The honeybees were surface-sterilized with 75% ethanol for 30 s, followed by three rinses with sterile deionized water to remove any ethanol residue. The honeybees were then placed on ice for dissection. Using sterile tweezers, the gut was carefully extracted by gently pulling from the base of the abdomen near the sting, ensuring the entire gut tissue was collected. The tissue was then placed in a 1.5 mL sterile centrifuge tube containing 1 × PBS. The gut tissues were then homogenized using a tissue grinder. The resulting homogenate was serially diluted, and 200 μL of the 10^−5^ and 10^−6^ dilutions were plated onto Tryptic Soy Agar (TSA, T8650, Solarbio, Beijing, China). The plates were incubated at 37 °C with 5% CO_2_ for 48 h. Single colonies were picked and transferred to Tryptic Soy Broth (TSB, LA0110, Solarbio, Beijing, China) for enrichment culture under the same conditions for enrichment. The resulting culture was then mixed with 50% glycerol at a 1:1 ratio and stored at −80 °C for future use.

For DNA extraction, 5 mL of bacterial culture in the logarithmic growth phase was collected, and bacterial pellets were harvested by centrifugation. Genomic DNA was extracted using a bacterial DNA extraction kit (D3350, Omega Bio-Tek, Norcross, GA, USA). DNA concentration and quality were assessed using a fluorescence detector (TBS-380, Turner BioSystems, Sunnyvale, CA, USA) and 0.8% agarose gel electrophoresis. Qualified DNA samples were used for subsequent sequencing library construction.

### 2.2. Library Construction and Illumina HiSeq Sequencing

Sequencing libraries were constructed using the genomic DNA of qualified strains, and paired-end sequencing (2 × 150 bp) was performed on the Illumina platform. The paired-end library, with an insert size of approximately 400 bp, was constructed following the standard protocol. Genomic DNA was first fragmented to the target size using a Covaris sonicator (M220, Woburn, MA, USA), and then T4 DNA polymerase was used to generate blunt ends. The 3′ ends of the phosphorylated blunt-ended fragments were added with an “A” base, and adapters were ligated to the DNA ends. The target fragments were purified by gel electrophoresis and selectively enriched and amplified by PCR. During PCR amplification, index tags were incorporated into the adapters, and the library quality was assessed. Finally, qualified paired-end libraries were sequenced using the Illumina Hiseq platform (PE150 mode, San Diego, CA, USA).

### 2.3. Genome Assembly

Raw sequencing data was generated by Illumina’s base calling software CASAVA v1.8.2 as outlined in the corresponding manuscript “https://support.illumina.com/sequencing/sequencing_software/casava.ilmn (accessed on 22 January 2025)”. Contamination reads, such as those containing adaptors or primers, were identified and removed by Trimmomatic [40] with default settings. The clean data, obtained after this quality control step, were then used for subsequent analyses. Genome assembly was conducted with ABySS, utilizing multiple-Kmer values to optimize the results [41]. To address remaining gaps and correct single base polymorphisms, GapCloser was applied to refine the final assembly [42].

### 2.4. Genome Annotation and Phylogenetic Construction

The Bakta command with default parameters was used to predict the structural and functional annotations [43]. tRNA were identified with tRNAscan-SE (v1.23) [44] while rRNA genes were detected using RNAmmer (v1.2) [45]. Mobile Genetic Elements (MGEs) were identified with mobileOG-db Beatrix-1.6, setting the E-value threshold to 1.0 × 10^−10^ [46]. Genome comparison was conducted using the OrthoFinder tool, with parameters set as Identity 70, Ratio (core) 0.95, and Support −1 [47]. KEGG was used for genome annotation, with a similarity threshold of 0.5 [48]. Phylogenetic analysis was performed using FastTree from VBCG software (v 1.3), with the max missing genes parameter set to 2 [49].

### 2.5. Colonization Experiments

Healthy brood combs used for rearing Germ-free worker bees were obtained from the experimental apiary at Zhejiang University. The method for obtaining germ-free worker bees was followed as previously described [28]. The procedure was as follows: Germ-free worker bee pupae were carefully extracted using sterile forceps and placed in a sterile 48-well cell culture plate. The plates were then incubated in an incubator at 32 ± 1 °C with 80 ± 5% relative humidity until the bees emerged (0 days). Newly emerged worker bees (0–48 h old) were subjected to a 3 h fasting period before being fed a sucrose solution containing bacterial suspension at OD_600_ = 1. In the cross-colonization experiment, worker bees were inoculated with strain sourced either from *A. mellifera* or *A. cerana* in two treatment groups, while the control group was fed sterile sucrose solution. For the competitive colonization experiment, strains from both *A. mellifera* and *A. cerana* were mixed in a 1:1 ratio and inoculated into the gut of worker bees from both species. Each group consisted of three honeybee cages, with each cage containing 30 worker bees. To meet the protein requirements of the worker bees, sterile rapeseed pollen was provided to all groups. Throughout the experiment, the sugar water was replaced daily, and honeybee samples were collected 7 days post-inoculation. The honeybees were then dissected using sterile forceps to remove the gut, which was stored at −80 °C for further analysis.

### 2.6. DNA Extraction from the Gut and Bacterial Quantification

Genomic DNA was extracted from honeybee gut tissues using the TIANamp Stool DNA Kit (DP328, Tiangen, Beijing, China) following the manufacturer’s instructions. DNA quality and integrity were assessed by 1% agarose gel electrophoresis. DNA concentration and purity were measured using a NanoDrop 2000 spectrophotometer (Thermo Fisher Scientific, Waltham, MA, USA). To quantify the abundance of bacteria originating from different honeybee species in the gut, specific primers were designed to target unique sequences associated with *Snodgrassella* strains from *A. cerana* and *A. mellifera*. The primers used for quantification were: AC-SNO (5′~3′): [F: TGTGGTAATGGTGGTATGAA], [R: CCGCTGTACGAATATAGAAC] for *A. cerana* derived *Snodgrassella*, and AM-SNO (5′~3′): [F: CTGGGCGTACATGCTATT], [R: CAATCTTGCCAGCCATATC] for *A. mellifera* derived *Snodgrassella*. These primers were used to assess the abundance of different *Snodgrassella* strains in the gut of both honeybee species. Bacterial copy numbers in the honeybee gut were quantified using a StepOne real-time quantitative PCR system (4376357, Thermo Fisher Scientific, Foster City, CA, USA). A standard curve between DNA concentration and CT value was established based on the method of Zhou et al. [30]. The qPCR amplification reaction was performed in a 10 μL volume, which included: 5 μL of 2 × SYBR Green Pro Taq HS (AG11701, Accurate Biology, Changsha, China), 1 μL of each 10 μM primer (Sangon, Shanghai, China), 2 μL of H_2_O, and 1 μL of template DNA. The qPCR cycling conditions were as follows: an initial denaturation at 95 °C for 30 s, followed by 40 cycles of denaturation at 95 °C for 30 s, and final annealing at 60 °C for 30 s. A melting curve analysis was conducted at the end of the amplification to verify primer specificity.

### 2.7. RNA Extraction, Immune Gene Expression, and Virus Titer Measurement

To investigate the effect of *Snodgrassella* strain colonization from different sources on honeybee transcriptional levels, RNA was isolated from worker bee samples 7 days after bacterial inoculation. Total RNA was extracted using the RNA extraction kit (AG11707, Accurate Biology, Changsha, China) according to the manufacturer’s protocol. Subsequently, cDNA was synthesized using a reverse transcription kit (AG11728, Accurate Biology, Changsha, China) and was used for subsequent qPCR analysis. The RT-qPCR procedure and reaction setup were consistent with the standard qPCR protocol outlined in Section 2.6. The relative expression levels of immune genes were calculated using the 2^(-ΔΔCt) method [38]. Primer sequences are provided in Table S2. Virus titers were determined by establishing standard curves for DWV (5′~3′): [F: ACCTGGAACATCAGGTAAGCG], [R: TTGAATCTCGAGTTCGGGACG] [32] and SBV (5′~3′): [F: AACGTCCACTACACCGAAATGTC], [R: ACACTGCGCGTCTAACATTCC] [50], and the absolute abundance of the viruses in the samples were calculated based on the Ct values.

### 2.8. Statistical Analysis

The phylogenetic tree of the genomic sequences was visualized using the tvBOT tool [51]. Statistical analysis was performed using GraphPad Prism software (8.0.2). One-way analysis of variance (ANOVA) followed by post-hoc multiple *t*-tests was used to assess differences in strain colonization levels, immune gene expression, and naturally occurring virus titers between groups. For each measurement, 6 samples per group were used to ensure the reproducibility and reliability of the experiment. A *p*-value of less than 0.05 was considered statistically significant.

## 3. Results

### 3.1. Snodgrassella Phylogenetic Trees Based on Core Gene Sequences Were Clustered by Host Type

**Figure 1:**
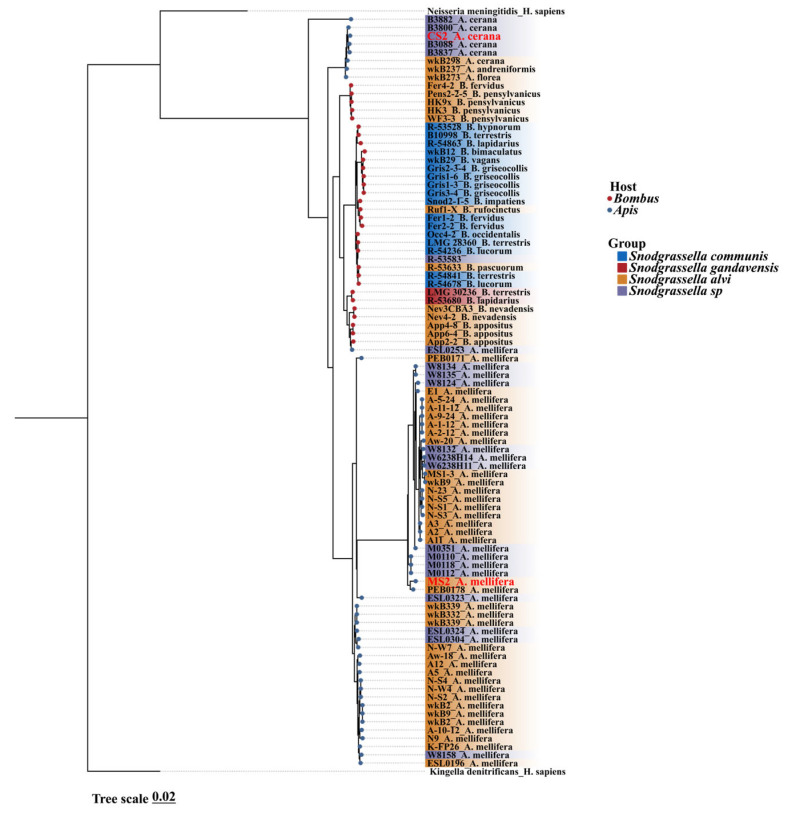
Maximum−likelihood phylogenies of 91 *Snodgrassella* strains, along with two outgroup strains (*Kingella denitrificans* and *Neisseria meningitidis*), were constructed based on core genes. The color blocks on the branches, represent the bacterial species classification. Blue and red circles at the branch nodes describes the genus of the host. Strains isolated in this study are highlighted in red text.

We constructed a phylogenetic tree using 89 *Snodgrassella* genomes sourced from the NCBI database, along with two strains isolated in this study. Additionally, we selected *Kingella denitrificans* (GCA_016127355.1) and *Neisseria meningitidis* (GCA_022869645.1) as outgroups for tree construction. Of these, 59 strains were from the *Apis* genus, with 51 originating from *A. mellifera*, 6 from *A. cerana*, and two from other *Apis* species. Additionally, 31 strains were derived from the *Bombus* genus, and one strain, R-53583, was of unknown origin. The inclusion criteria for genome selection were genome completeness greater than 90% and contamination levels below 5%. The phylogenetic inference for *Snodgrassella* was constructed based on core genes. The strains in the phylogenetic tree predominantly clustered according to their host species. Our *A. mellifera* derived isolate, strain MS2, clustered within the *S. alvi* clade, whereas the *A. cerana* derived strain, CS2, grouped within an unclassified *Snodgrassella* clade (Figure 1). Notably, within the *Snodgrassella* phylogeny, strains from the same host species typically clustered together. Strains derived from *A. cerana* and *Bombus* species formed two sister clades, which were in turn sister to the *Snodgrassella* strains of *A. mellifera*.

### 3.2. Genome Characteristics of Snodgrassella

**Figure 2:**
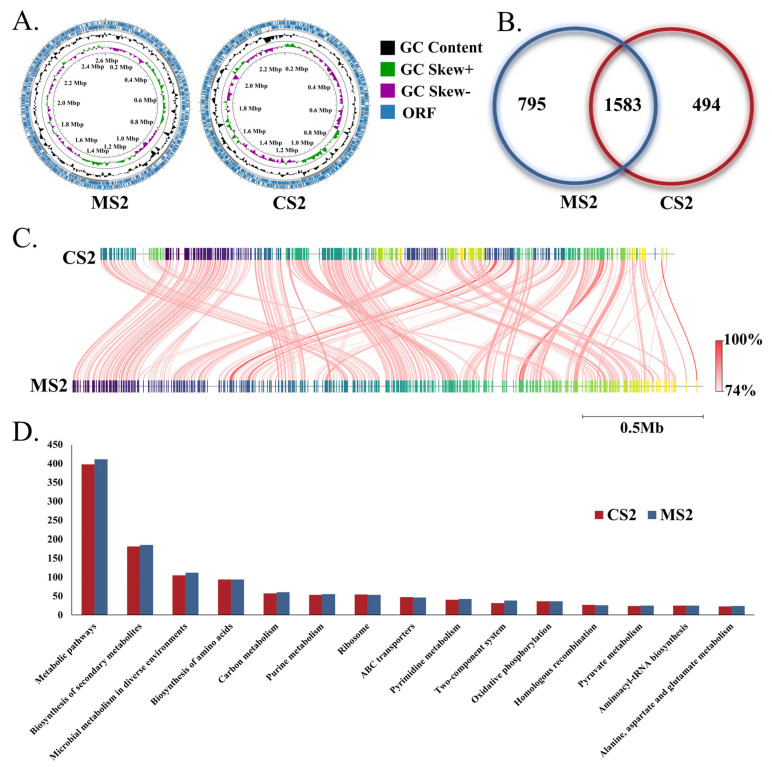
Genomic structures of *Snodgrassella* strains derived from *A. cerana* and *A. mellifera*. (**A**) Schematic representation and statistical overview of *Snodgrassella* genomes sequenced from *A. cerana* and *A. mellifera*. (**B**) Numbers of shared orthologous genes and unique genes in *Snodgrassella* strains from *A. cerana* and *A. mellifera*. (**C**) Average nucleotide identity (ANI) of *Snodgrassella* genomes derived from *A. cerana* and *A. mellifera*. (**D**) Gene content categorized by the Kyoto Encyclopedia of Genes and Genomes (KEGG) subsystems. MS2: *A. mellifera* derived *Snodgrassella* strain; CS2: *A. cerana* derived *Snodgrassella* strain.

The genomes of the isolated strains CS2 and MS2 were assembled into 34 and 39 scaffolds, respectively, resulting in genome sizes of 2,386,043 bp and 2,621,985 bp (Figure 2A). The CS2 genome encodes 2077 proteins, 55 tRNAs, and 3 rRNA operons, while the MS2 genome encodes 2378 proteins, 57 tRNAs, and 4 rRNA operons (Figure 2B and Table S1). The MS2 genome is larger than that of CS2, with a higher number of protein-coding genes, non-coding RNAs (ncRNAs), and mobile genetic elements (Table S1). The average nucleotide identity (ANI) values among the 91 *Snodgrassella* genomes ranged from 73.58% to 99.99% (Figure S1), with a ANI of 79.83% between the genomes of CS2 and MS2 (Figure 2C). KEGG annotation revealed that the two strains share a similar functional profile, with genes primarily enriched in categories such as metabolic pathways, biosynthesis of secondary metabolites, microbial metabolism in diverse environments, biosynthesis of amino acids, and carbon metabolism (Figure 2D). Notably, MS2 exhibited a higher number of genes enriched in these major functional categories compared to CS2 (Figure 2D).

### 3.3. Gut Colonization of Snodgrassella Strains

**Figure 3:**
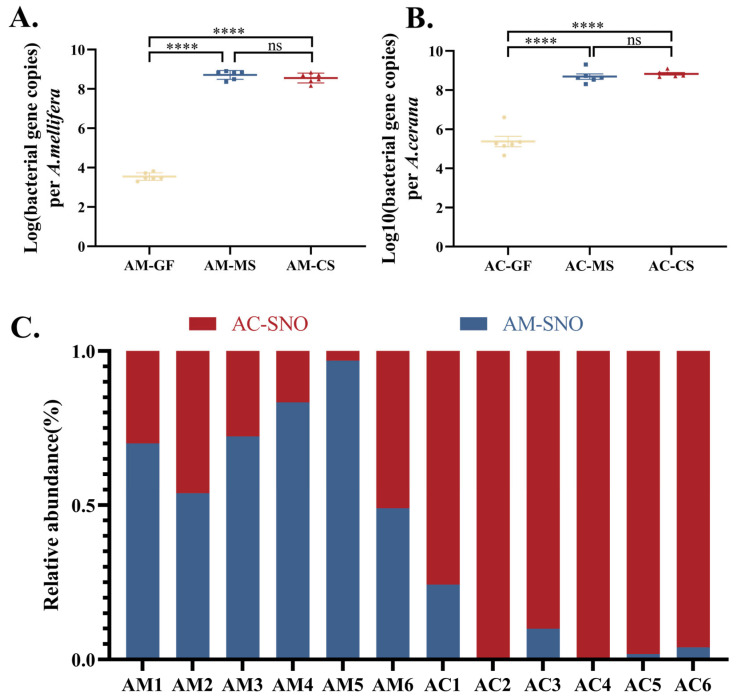
Colonization of *Snodgrassella* strains from *A. cerana* and *A. mellifera* in honeybee gut (each group contains 6 samples). (**A**) Colonization of *Snodgrassella* strain from *A. cerana* or *A. mellifera* in the gut of *A. mellifera* workers. (**B**) Colonization of *Snodgrassella* strain from *A. cerana* or *A. mellifera* in the gut of *A. cerana* workers. (**C**) Competitive colonization of *Snodgrassella* strains from *A. cerana* and *A. mellifera* in honeybee gut. The horizontal line on the graph represents the mean of each group, while the whiskers indicate the standard error of the mean (SEM) for each group. Significance levels are marked by different symbols: ****, *p* < 0.0001; ns, not significant. AM-GF: Germ-free *A. mellifera* workers (control group). AM-MS: *A. mellifera* workers inoculated with *A. mellifera* derived *Snodgrassella* strain. AM-CS: *A. mellifera* workers inoculated with *A. cerana* derived *Snodgrassella* strain. AC-GF: Germ-free *A. cerana* workers (control group). AC-MS: *A. cerana* workers inoculated with *A. mellifera* derived *Snodgrassella* strain. AC-CS: *A. cerana* workers inoculated with *A. cerana* derived *Snodgrassella* strain. AC-SNO: *A. cerana* derived *Snodgrassella* strain. AM-SNO: *A. mellifera* derived *Snodgrassella* strain.

To explore the physiological basis of host-specific evolution, *Snodgrassella* strains from *A. cerana* and *A. mellifera* were inoculated into the gut of germ-free worker bees of the corresponding species, which were reared in the laboratory. The bacterial load in the germ-free worker bees was less than 10^5^, confirming the successful establishment of the germ-free honeybee model (Figure 3A,B). The results from the cross-colonization experiment demonstrated that both *A. cerana* and *A. mellifera* strains were able to successfully colonize the gut of honeybees from different host species, with colonization levels reaching 10^8^ copies (Figure 3A,B). Although no significant difference in colonization levels was observed between native and non-native strain in both *A. cerana* and *A. mellifera* gut (Figure 3A,B), the competitive colonization experiment showed that native strain exhibited a stronger colonization advantage in the honeybee gut (Figure 3C).

### 3.4. Colonization of Native Snodgrassella Strain Reduces Naturally Occurring Virus Titers in A. mellifera

**Figure 4:**
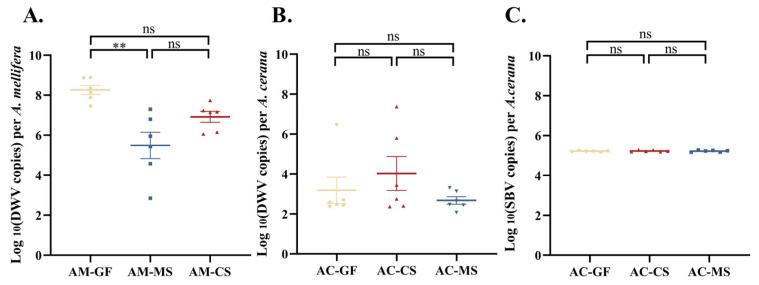
Naturally occurring virus titers in honeybees following colonization with *Snodgrassella* strains from *A. cerana* and *A. mellifera* (each group contains 6 samples). (**A**) Naturally occurring DWV titers in *A. mellifera* following colonization with *Snodgrassella* strain from *A. cerana* or *A. mellifera*. (**B**) Naturally occurring DWV titers in *A. cerana* following colonization with *Snodgrassella* strain from *A. mellifera* or *A. cerana*. (**C**) Naturally occurring SBV titers in *A. cerana* following colonization with *Snodgrassella* strain from *A. mellifera* or *A. cerana*. The horizontal line on the graph represents the mean of each group, while the whiskers indicate the SEM for each group. Significance levels are marked by different symbols: **, *p* < 0.01; ns, not significant. AM-GF: Germ-free *Apis mellifera* workers (control group). AM-MS: *A. mellifera* workers inoculated with *A. mellifera* derived *Snodgrassella* strain. AM-CS: *A. mellifera* workers inoculated with *A. cerana* derived *Snodgrassella* strain. AC-GF: Germ-free *A. cerana* workers (control group). AC-MS: *A. cerana* workers inoculated with *A. mellifera* derived *Snodgrassella* strain. AC-CS: *A. cerana* workers inoculated with *A. cerana* derived *Snodgrassella* strain.

Viral titers naturally produced in the host following colonization with native and non-native *Snodgrassella* strains were measured. Compared to the germ-free control group, the MS2 treatment significantly reduced DWV titers in *A. mellifera*, whereas the CS2 treatment had no significant effect on DWV titers (Figure 4A). In contrast, for *A. cerana*, inoculation with either MS2 or CS2 did not result in significant changes in the titers of SBV or DWV (Figure 4B,C). Notably, regardless of treatment or the origin of the *Snodgrassella* strain, the natural DWV titers in *A. cerana* were consistently lower than those in *A. mellifera* under the same experimental conditions. (Figure 4B,C).

### 3.5. Colonization of Native Snodgrassella Strain Promotes the Expression of the Defensin 2 Gene in A. mellifera

**Figure 5:**
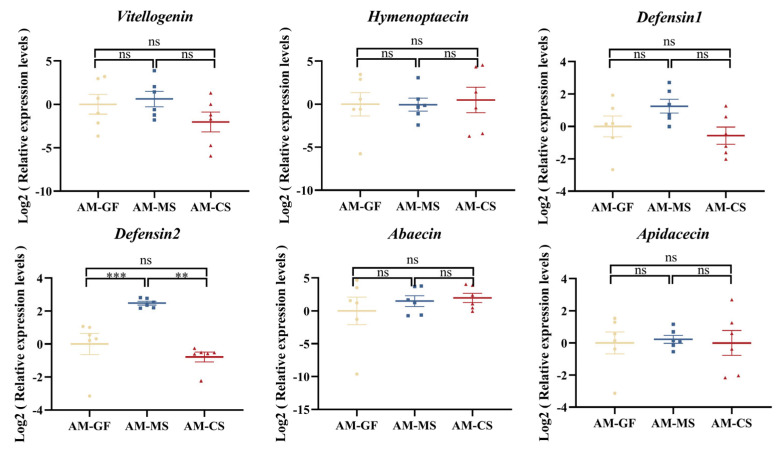
Expression of immune−related genes in *A. mellifera* following colonization with *Snodgrassella* strains from *A. cerana* and *A. mellifera* (each group contains 6 samples). The horizontal line on the graph represents the mean of each group, while the whiskers indicate the SEM for each group. Significance levels are marked by different symbols: **, *p* < 0.01; ***, *p* < 0.001; ns, not significant. AM-GF: Germ-free *A. mellifera* workers (control group). AM-MS: *A. mellifera* workers inoculated with *A. mellifera* derived *Snodgrassella* strain. AM-CS: *A. mellifera* workers inoculated with *A. cerana* derived *Snodgrassella* strain.

**Figure 6:**
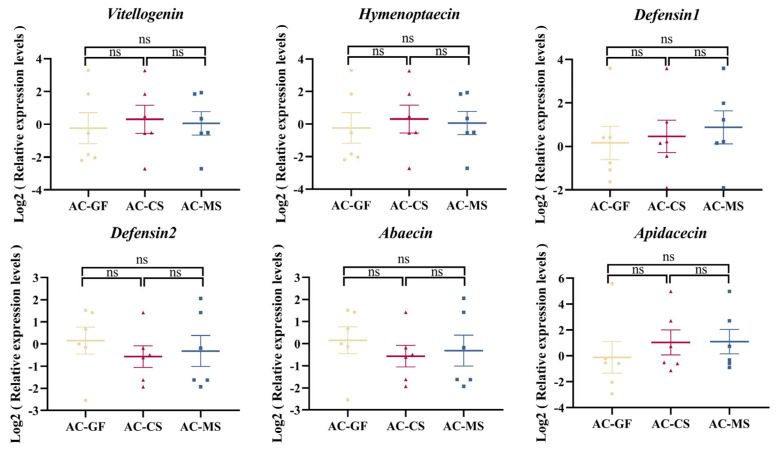
Expression of immune-related genes in *A. cerana* following colonization with *Snodgrassella* strains from *A. cerana* and *A. mellifera* (each group contains 6 samples). The horizontal line on the graph represents the mean of each group, while the whiskers indicate the SEM for each group. Significance levels are marked by different symbols: ns, not significant. AC-GF: Germ-free *A. cerana* workers (control group). AC-MS: *A. cerana* workers inoculated with *A. mellifera* derived *Snodgrassella* strain. AC-CS: *A. cerana* workers inoculated with *A. cerana* derived *Snodgrassella* strain.

To investigate whether the effect of *Snodgrassella* colonization on host immunity varies depending on the strain origin, we measured the expression levels of immune-related genes in the host after stable colonization of the gut with *Snodgrassella* strains from different sources. The results showed that, in *A. mellifera*, inoculation with MS2 significantly increased the expression of *defensin 2* gene compared to both the control group and the CS2 group (Figure 5). However, in *A. cerana*, no significant changes in immune gene expression were observed, regardless of whether the strain was native or non-native (Figure 6).

## 4. Discussion

Revealing the interactions between animal hosts and their symbiotic microbiota in different animal models helps enhance our understanding of the diversity of host-symbiont relationships and the evolutionary history of these interactions. The geographic isolation of *A. cerana* and *A. mellifera* for millions of years has limited the potential interactions between their gut microbiota, allowing for the development of specialized gut microbiomes that co-evolved with their respective hosts [52]. In this study, we isolated *Snodgrassella* strains from the gut microbiota of both honeybee species and conducted colonization experiments to explore the host specificity of *Snodgrassella* strains and their underlying physiological and functional basis. Our results confirm the evolution of gut microbiome host specificity within species and reveal the impact of strain-level variation on differences in host physiological functions.

Phylogenetic analysis of existing *Snodgrassella* strains based on core genes revealed that *Snodgrassella* strains from *A. cerana* and *A. mellifera* formed distinct branches, indicating extensive genomic differences between species. Interestingly, the phylogeny of *Snodgrassella* strains did not align with host phylogeny, which is consistent with previous studies based on gene flow trees [13]. However, recent metagenomic sequencing based on the core genome of *Snodgrassella* showed a phylogenetic pattern that is consistent with the host phylogeny [53]. This discrepancy may be attributed to occasional host-switching events, disrupting the co-diversification between symbionts and their hosts [21,54]. The ANI values between the genomes of *Snodgrassella* strains still exceed 95%, suggesting that finer taxonomic distinctions are needed for classifying bee gut microbiota. Consistent with previous metagenomic sequencing results of *A. cerana* and *A. mellifera* [28,29], our genomic analysis of individual strains reveals that the *A. mellifera* isolates have larger genomes, higher gene content, and a greater number of MGEs. This may be related to the translocation and expansion of *A. mellifera* populations outside their native range, which requires larger genomes and functional genes to rapidly adapt to changing environments. Variations in the genes of gut microbiota and their functional differentiation are key drivers of host adaptive evolution and provide the physiological and functional basis for host-specific colonization [13]. Previous studies on the host specificity of gut microbiota in *A. mellifera* and bumblebees have shown that native strains exhibit a distinct competitive advantage in colonization experiments [12,20,21,22,23,24]. In our study, we similarly observed a competitive advantage of native strain in the gut of both *A. cerana* and *A. mellifera*. These results suggest that even among closely related species within the same genus, gut microbiota exhibit host specificity, forming the physiological and functional foundation for host-specific colonization.

The gut microbiota plays a crucial role in defending honeybees against pathogen invasion, including resistance to opportunistic bacteria, fungal pathogens, and RNA viruses [55,56,57,58]. When honeybees are exposed to DWV, those lacking a microbiome exhibit significantly lower survival rates compared to normal honeybees [56]. Antibiotic-induced disruption of the gut microbiota reduces honeybee resistance to IAPV, increasing susceptibility [59,60]. Additionally, pathogen-induced dysbiosis in the gut promotes the proliferation of Chronic Bee Paralysis Virus in the honeybee gut, leading to higher mortality [61]. Studies have shown that *S. alvi* inoculation can improve honeybee survival under DWV infection [39]. Based on the protective role of *S. alvi* against host pathogens, plasmid-modified *S. alvi* has been developed to combat honeybee pathogens such as *Microsporidia* and DWV through RNA interference [57,62]. In this study, we found that inoculation with native *Snodgrassella* strain significantly reduced the naturally occurring DWV titers in *A. mellifera*, whereas inoculation with non-native strain did not significantly alter the virus titers. This highlighting the importance of host specificity in symbiotic systems. These results further confirm the protective role of *Snodgrassella* in defending *A. mellifera* from pathogen invasion and underscore the impact of strain-level variation on host physiological functions. However, regardless of whether native or non-native *Snodgrassella* strains were inoculated, there was no significant change in naturally occurring DWV titers in *A. cerana*. This indicates a more complex interaction between host populations, microbiota, and virus adaptability. In *A. cerana*, DWV typically exhibits lower virulence, and the virus-host relationship appears to be more stable [63]. In contrast, in *A. mellifera*, the virus is more virulent due to cross-species transmission via *V. destructor* [64]. Initially, the DWV-A virus, originating in the ancestral host *A. cerana*, is transmitted to *A. mellifera* by *V. destructor*, leading to an increase in viral virulence and posing a significant threat to *A. mellifera* populations [65]. Notably, using universal primers for DWV, we observed that naturally occurring DWV titers in *A. mellifera* were higher than in *A. cerana* under the same experimental conditions. Under symptomless viral load limits, the presence of the core gut microbiota *Snodgrassella* in honeybees seemed to play a role in reducing higher viral titers of naturally occurring DWV. In contrast, *Snodgrassella* had minimal effect on lower viral titers. Similarly, inoculation with *Snodgrassella* did not significantly affect the naturally occurring SBV levels in honeybees, which exhibited lower viral titers. Therefore, the beneficial effects of *Snodgrassella* colonization in reducing naturally occurring DWV titers may be more pronounced in *A. mellifera* than in *A. cerana*. These findings suggest that, during seasons with high *V. destructor* infection rates, inoculation with native *Snodgrassella* strains could be a potential strategy to control sudden DWV outbreaks by reducing the viral load in asymptomatically infected honeybees. This could help mitigate the synergistic effects of DWV and *V. destructor* on colony health. These findings align with previously established roles of probiotics in honeybee colonies. For instance, supplementing colonies with the probiotic LX3 (a mixture of *Lactiplantibacillus plantarum* Lp39, *Lacticaseibacillus rhamnosus* GR-1, and *Apilactobacillus kunkeei* BR-1), derived from honeybee gut isolates, has been shown to more effectively reduce pathogen loads compared to oxytetracycline treatment [66]. Furthermore, host-derived strains, such as *Lactobacillus kunkeei* and *Lactiplantibacillus plantarum*, provide protective effects against chalkbrood infections [67,68]. Collectively, these results underscore the potential of host-derived bacterial strains as probiotics, enhancing the colony’s resilience to multiple stressors.

The gut microbiota may influence honeybee health by modulating host immune response [69,70,71]. Colonization by the microbiota or its individual members can upregulate the expression of host immune genes, providing protection against opportunistic bacteria, virus, fungi, and parasites [72,73,74,75]. *S. alvi* colonization can promote the upregulation of host genes encoding antimicrobial peptides [27]. In this study, we also observed that inoculation with native *Snodgrassella* strain significantly upregulated the expression of the *defensin 2* gene in *A. mellifera*, while inoculation with non-native strain did not induce significant changes in immune gene expression. In contrast, in *A. cerana*, no significant changes in immune gene expression were observed, regardless of whether the strain was native or non-native. This suggests that the immunomodulatory effects of *Snodgrassella* strains are host-specific in *A. mellifera*. The expression of immune genes in *A. cerana* further confirmed that the host or gut microbiota response is limited when the viral titers are at low copy numbers. The upregulation of *defensin 2* gene level in *A. mellifera* following *Snodgrassella* inoculation may be one of the mechanisms through which *Snodgrassella* reduces naturally occurring DWV titers. However, the molecular mechanisms underlying these effects require further investigation. Although *A. cerana* and *A. mellifera* are closely related species, we observed differences in their gut *Snodgrassella* strains roles in immune regulation and virus defense. These findings highlight the species-specific nature of the gut microbiome’s effects on host immunity and underscore the importance of further investigating the interactions between *A. cerana* gut microbiota and its host.

## 5. Conclusions

Our study demonstrates that *Snodgrassella* strains in *A. cerana* and *A. mellifera* exhibit host-specific genomic adaptations and colonization advantages, which directly influence host health. In *A. mellifera*, native *Snodgrassella* inoculation reduced DWV loads and upregulated the expression of *defensin 2*, suggesting a protective role against viral proliferation. These findings propose a new biological control strategy: applying native *Snodgrassella* to asymptomatically DWV-infected colonies can reduce sudden outbreaks and spread of DWV. This suggests that strain specificity should be carefully considered when developing honeybee probiotic formulations. Future work should optimize strain delivery methods and evaluate field efficacy, offering a sustainable tool to enhance pollinator health and ecosystem resilience.

## Data Availability

The original contributions presented in this study are included in the article/Supplementary Materials. The genomes of the isolates are deposited in the NCBI under BioProject PRJNA1256677. Further inquiries can be directed to the corresponding author.

## Supplementary Materials

The following supporting information can be downloaded at: https://www.mdpi.com/article/10.3390/insects16050478/s1. Figure S1: The ANI values between the genomes of 91 strains under the *Snodgrassella* genus; Table S1: Genomic characteristics of 2 *Snodgrassella* strains; Table S2: Immune gene primers.
