# Supplementary material for: Host Specificity of Snodgrassella in Eastern and Western Honeybees and Its Effects on Naturally Occurring Deformed Wing Virus Titers
Source: Insects. 2025 May 1;16(5):478. doi: 10.3390/insects16050478 (PMC12111971; doi:10.3390/insects16050478)
Supplement: Supplementary file 1 [file insects-16-00478-s001.zip › insects-3534403-supplementary.pdf]

Supporting Information for

Host Specificity of *Snodgrassella* in Eastern and Western Honeybees and Its Effects on Naturally Occurring Deformed Wing Virus Titers

Table S1. Genomic characteristics of 2 *Snodgrassella* strains.

Genomic characteristics	MS2 (*Apis mellifera*)	CS2 (*Apis cerana*)
Sequence size(bp)	2,621,985	2,386,043
Number of contigs	39	34
GC content (%)	41	43
Protein coding genes	2378	2077
rRNA operons	4	3
tRNAs	57	55
Mobile element genes	65	50

Table S2. Immune gene primers.

**Gene name**	**Gene category**	**Primer sequeces (5′to 3′)**
Abaecin	Immune	F: AGATCTGCACACTCGAGGTCTG
		R: TCGGATTGAATGGTCCCTGA
Apidacecin	Immune	F: TAGTCGCGGTATTTGGGAAT
		R: TTTCACGTGCTTCATATTCTTCA
Hymenoptaecin	Immune	F: ATATCCCGACTCGTTTCCGA
		R: TCCCAAACTCGAATCCTGCA
defensin-1	Immune	F: TGTCGGCCTTCTCTTCATGG
		R: TGACCTCCAGCTTTACCCAAA
defensin-2	Immune	F: GCAACTACCGCCTTTACGTC
		R: GGGTAACGTGCGACGTTTTA
eater	Immune	F: CATTTGCCAACCTGTTTGT
		R: ATCCATTGGTGCAATTTGG
Actin	Housekeeping	F: ATGCCAACACTGTCCTTTCTGG
		R: GACCCACCAATCCATACGGA

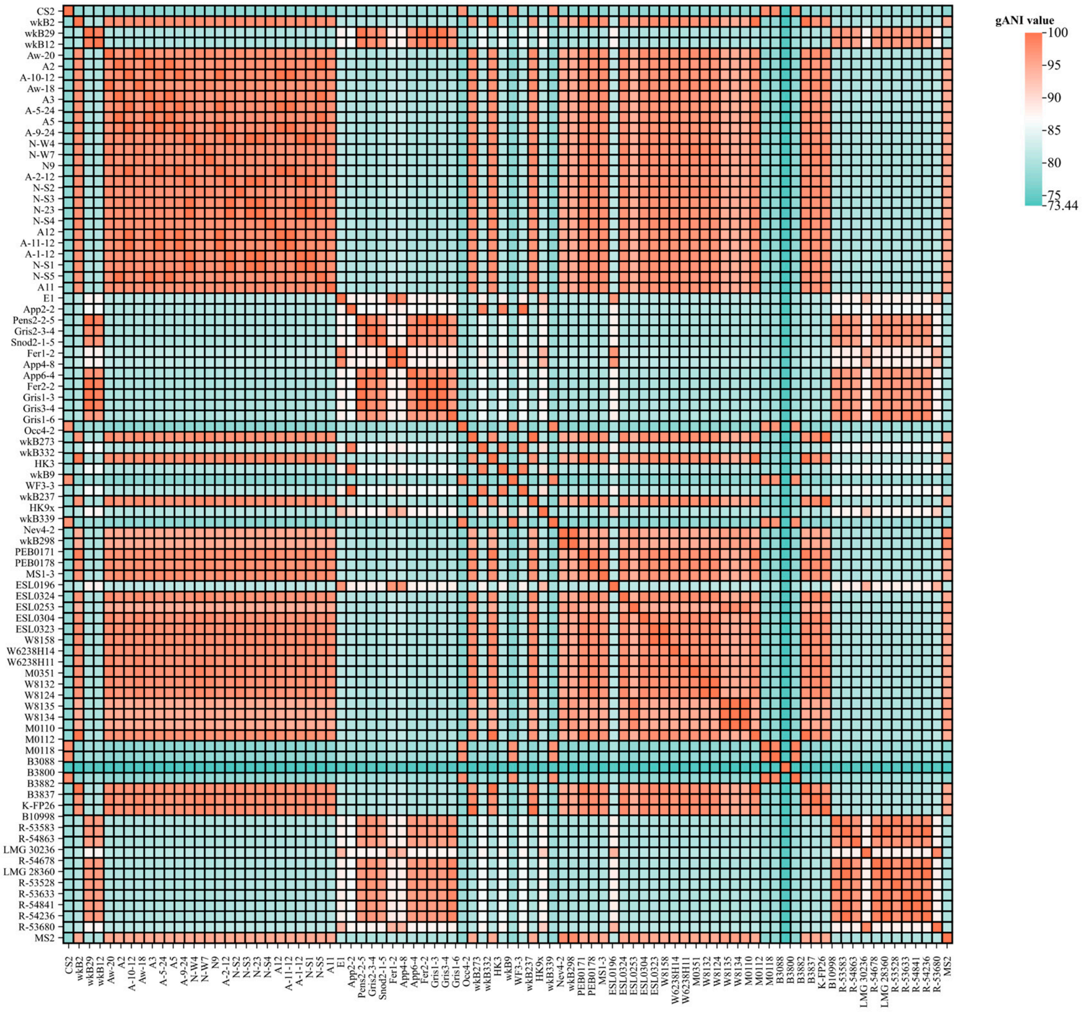

Figure S1. The ANI values between the genomes of 91 strains under the *Snodgrassella* genus.
